# Assessment of HPV screening modalities within primary care: a systematic review

**DOI:** 10.3389/fmed.2025.1567509

**Published:** 2025-04-09

**Authors:** Yahya Mostafa Waly, Abu-Baker Sharafeldin, Abdulrahman Al-Majmuei, Mohammad Alatoom, Salim Fredericks, Adri-Anna Aloia

**Affiliations:** ^1^School of Medicine, Royal College of Surgeons in Ireland, Medical University of Bahrain, Al Muharraq, Bahrain; ^2^Foundation of International Medical Graduates, Toronto, ON, Canada

**Keywords:** self-sampling, human papillomavirus (HPV), sensitivity, specificity, patient preference

## Abstract

**Introduction:**

Most cervical cancer precancerous lesions are associated with high-risk human papillomavirus (HPV) subtypes. Early detection through screening is crucial for preventing and managing HPV-related diseases. HPV Self-sample screening is a proposed method that can mitigate socioeconomic disparities, reduce embarrassment and costs of screening. This can possibly reduce the overall disease burden.

**Methods:**

A search strategy was conducted across multiple databases, including PubMed, Cochrane Library, Scopus, and Embase. Data extraction was performed using a standardized form to collect detailed information on study characteristics, participant demographics, and various outcomes. The quality and risk of bias in the articles were assessed using the Critical Appraisal skills programme (CASP) checklist, and the Cochrane Risk of Bias (ROB) tool.

**Results:**

Our review consistently found that HPV self-sampling is comparable to clinician-collected samples in terms of HPV detection rates and sensitivity, supporting the idea that HPV self-sampling can be a viable alternative for cervical cancer screening. Across the studies, self-sampling showed comparable or greater effectiveness to clinician-collected samples in detecting HPV in individuals. Specificity was comparable between both methods, with clinician-collected sampling slightly outperforming HPV self-sampling in some cases. Moreover when analyzing the negative predictive value (NPV) and positive predictive value (PPV) across the studies, it was evident that there was little difference between clinician-collected sampling and HPV self-sampling. 64.3% favored self-sampling over clinician-collected sampling due to increased comfort and privacy. Overall, the evidence suggests that self-sampling is an effective, patient-preferred, and cost-efficient alternative to clinician-collected sampling, particularly in under-screened populations.

## Introduction

Human Papillomavirus (HPV) remains a significant public health concern, being the most common sexually transmitted infection globally and a primary cause of cervical cancer (1). Early and accurate detection through screening is crucial for preventing and managing HPV-related diseases. As reported by the World Health Organization (WHO), approximately 660,000 new cervical cancer cases were recorded in 2022, making it the fourth most prevalent cancer among women (2). Traditionally, cervical cancer screening has relied on clinician-collected samples, where a healthcare provider collects a cervical sample using a speculum and cytobrush or spatula for testing. This method, often performed in a clinical setting, has been the standard for detecting high-risk HPV types and cervical precancerous lesions. However, self-sample screening has recently gained attention as a viable alternative, particularly in primary care settings, due to its ease of use and accessibility. Research from the Centers for Disease Control and Prevention (CDC) indicates that high-risk HPV types are found in 99% of cervical precancers, highlighting the need for effective screening methods (3). Self-sample screening allows women to collect their samples, which can enhance comfort and privacy and reduce barriers to screening participation. The incidence of cervical cancer reveals significant socioeconomic disparities that affect the disease’s burden (4). Addressing these disparities through accessible screening methods could potentially improve health outcomes for underserved populations by increasing screening participation and early detection. Despite these potential benefits, it is essential to determine whether self-sampling methods are as clinically effective as clinician-collected samples at detecting HPV. Therefore, the insights gained from this comparative analysis will be valuable for healthcare providers and policymakers, potentially shaping future screening practices and enhancing efforts to prevent cervical cancer.

The decision for women to undergo cervical screening is multifactorial. Preference is one of the main factors affecting decision-making for women when it comes to screening for HPV. Women around the world tend to reject the screening test purely due to embarrassment (5). An example is shown in an article written in Japan, illustrating that approximately 58% of women who underwent the trial preferred self-sampling to physician-led sampling, purely based on the embarrassment factor (6). Lack of knowledge is another determinant, especially in areas where screening is not given attention or not made available to the population. A US article assessing reasons for not screening found lack of knowledge was the most common reason for those who have not received timely screening, i.e., 64.4% of Hispanic women (7). Women with higher education (college) were found to have a higher prevalence of screening than those who were still in high school in the United States (8). Cost is a crucial component that determines whether a woman should go for screening. In the US, women with no health insurance at all had a 17% lower prevalence of cervical screening than those with some form of insurance; a 6% increase in the prevalence of screening among women not avoiding care compared to women who avoid it purely due to cost (8). All these aspects and more assimilate to the lack of screening and cervical cancer being among the four most prevalent cancers among women (2). This systematic review aims to incorporate these components, to review, compare, and assess the present HPV screening modalities.

## Methodology

This systematic review was conducted in accordance with the Preferred Reporting Items for Systematic Reviews and Meta-Analyses (PRISMA) guidelines to ensure transparency and comprehensiveness, although the review protocol was not registered prior to the study.

Studies were selected based on the following inclusion criteria: Articles written in the English language and published within the last 10 years (2014–2024), female participants aged 18 years and older, and studies that directly compare the efficacy of HPV screening via self-sampling and clinician-collected sampling. Eligible study designs included original research, systematic reviews, and meta-analyses. The exclusion criteria included studies with participants younger than 18 years of age, articles not written in the English language, publications before 2014, literature reviews, gray literature, opinion pieces, and case series with less than 10 participants.

A comprehensive search strategy was conducted across multiple databases, including PubMed, Cochrane Library, Scopus, and Embase. The search used a combination of MeSH terms and keywords, with Boolean operators used to refine the search results and ensure a broad yet focused collection of relevant studies.

For PubMed, the search query included terms such as (“Papillomavirus Infections” [MeSH] OR “Human papillomavirus” OR “HPV testing” OR “HPV DNA testing”) AND (“Self Care” [MeSH] OR “Self-sampling” OR “Self-collection” OR “Self-testing” OR “Home testing”) AND (“Papanicolaou Test” [MeSH] OR “Pap smear” OR “Cytology” OR “Pap test” OR “Cervical cytology”) AND (“Sensitivity and Specificity” [MeSH] OR “Efficacy” OR “Accuracy” OR “Performance” OR “Diagnostic accuracy”) AND (“Comparison” OR “Versus” OR “Compared to” OR “Compared with” OR “Comparative”), yielding 80 results after applying filters.

In the Cochrane Library, the same advanced search was conducted, resulting in 23 Cochrane reviews after excluding 4,505 trials. The Scopus search retrieved 23 results, and the EMBASE search yielded 7,347 articles, with only 36 being considered for inclusion. This resulted in 162 total articles from each of the 4 databases being exported to the Rayyan web app where 3 independent reviewers (AA, YW, and MA) screened the titles and abstracts resolving differences in study selection through discussion.

Data extraction was performed using a standardized form to collect detailed information on study characteristics, participant demographics, and various outcomes. The primary outcomes included the detection rate of HPV in the self-sampling group compared to the clinician-collected sampling group, with comparative statistics such as odds ratios, relative risks, and statistical significance being assessed where possible. Secondary outcomes focused on the sensitivity and specificity of self-sampling versus clinician-collected sampling, the positive predictive value (PPV) and negative predictive value (NPV) of both methods, patient preference, cost-effectiveness, and the time required for patients to receive their results. Extractions were carried out independently by the same three reviewers, who also cross-checked the data for accuracy. It should be noted that not all outcomes were reported by the authors of the included studies.

Extracted studies reported HPV test results in varying formats. The majority of studies provided binary outcomes (positive or negative), while some also reported partial genotyping, identifying specific high-risk HPV types such as HPV 16 and HPV 18, or grouped into categories (e.g., high-risk HPV vs. low-risk HPV). Studies that performed partial genotyping typically used polymerase chain reaction (PCR)-based assays or hybrid capture technology, allowing for differentiation between individual HPV types. Additionally, the majority of studies utilized commercially validated HPV detection assays, including Hybrid Capture 2 (HC2), Cobas 4,800 HPV Test, APTIMA HPV Assay, Xpert HPV Test, and CareHPV Test. These assays have been widely recognized and approved for clinical use by regulatory bodies such as the U.S. Food and Drug Administration (FDA) and the European Medicines Agency (EMA). Additionally, some studies specified the use of in-house PCR assays, which underwent internal validation protocols to ensure reliability and reproducibility.

The quality and risk of bias in the articles were assessed using the Critical Appraisal Skills Programme (CASP) checklist, and the Cochrane Risk of Bias (ROB) tool. These tools were chosen due to their robust framework for evaluating the validity and relevance of the included studies. The CASP checklist provides a structured approach to assessing various study designs, making it particularly effective for the diverse types of studies included in our review. The Cochrane ROB tool was also selected as it is the gold standard for assessing bias in randomized controlled trials, of which there are several in our review. We did not opt for other assessments such as the Newcastle–Ottawa scale (NOS), or the GRADE approach as they were deemed to be less effective at addressing our concerns regarding validity and bias when compared to the former two.

## Results

### Literature search

**Figure 1:**
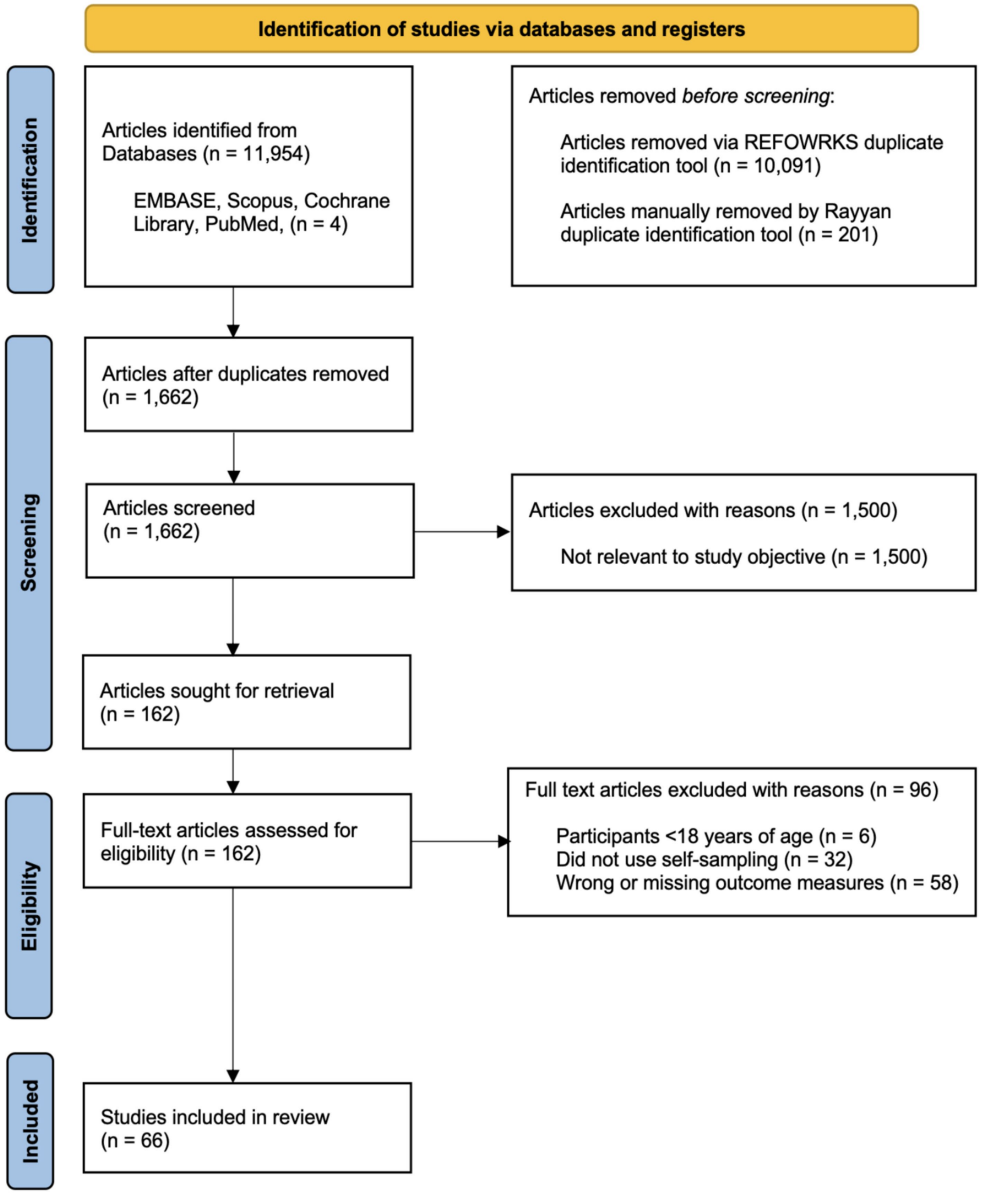
PRISMA 2020 flow diagram for systematic review screening and selection. Adapted from Page et al. (9).

A total of 11,954 articles were identified through an initial database search. Following title and abstract screening, 162 full-text articles were assessed for eligibility. Of these, 66 studies met the inclusion criteria and were included in the systematic review (Figure 1).

### Methods of assessment

The quality and risk of bias in the included studies were assessed using the Critical Appraisal Skills Programme (CASP) checklist and the Cochrane Risk of Bias (ROB) tool. These tools were chosen for their structured and comprehensive approach to evaluating study validity. The CASP checklist was particularly useful for assessing the diverse study designs included in this review, while the Cochrane ROB tool provided a standardized framework for evaluating randomized controlled trials. Of the 66 included studies, 54 (81.8%) demonstrated a low risk of bias across key areas, indicating strong methodological rigor. However, 12 studies (18.2%) had a moderate risk of bias due to factors such as small sample sizes, specific settings, and potential biases related to self-selection or test performance variability. While the overall findings remain reliable, these limitations should be considered when interpreting the results.

### Study characteristics

The 66 included studies comprised a total of 702,785 participants (excluding 4 systematic review/meta-analysis studies, 1 cost-effectiveness study, and 1 review study based on systematic reviews), with sample sizes ranging from 30 to 487,015 participants. The studies were conducted across 29 different countries, with the majority (43 studies) being cross-sectional studies. The remaining studies included 2 systematic reviews, 2 meta-analysis studies, 5 cohort studies, 10 randomized controlled trials, and 4 other studies, such as observational studies, comparative studies, and cost-effectiveness analyses.

### Study purpose

**Figure 2:**
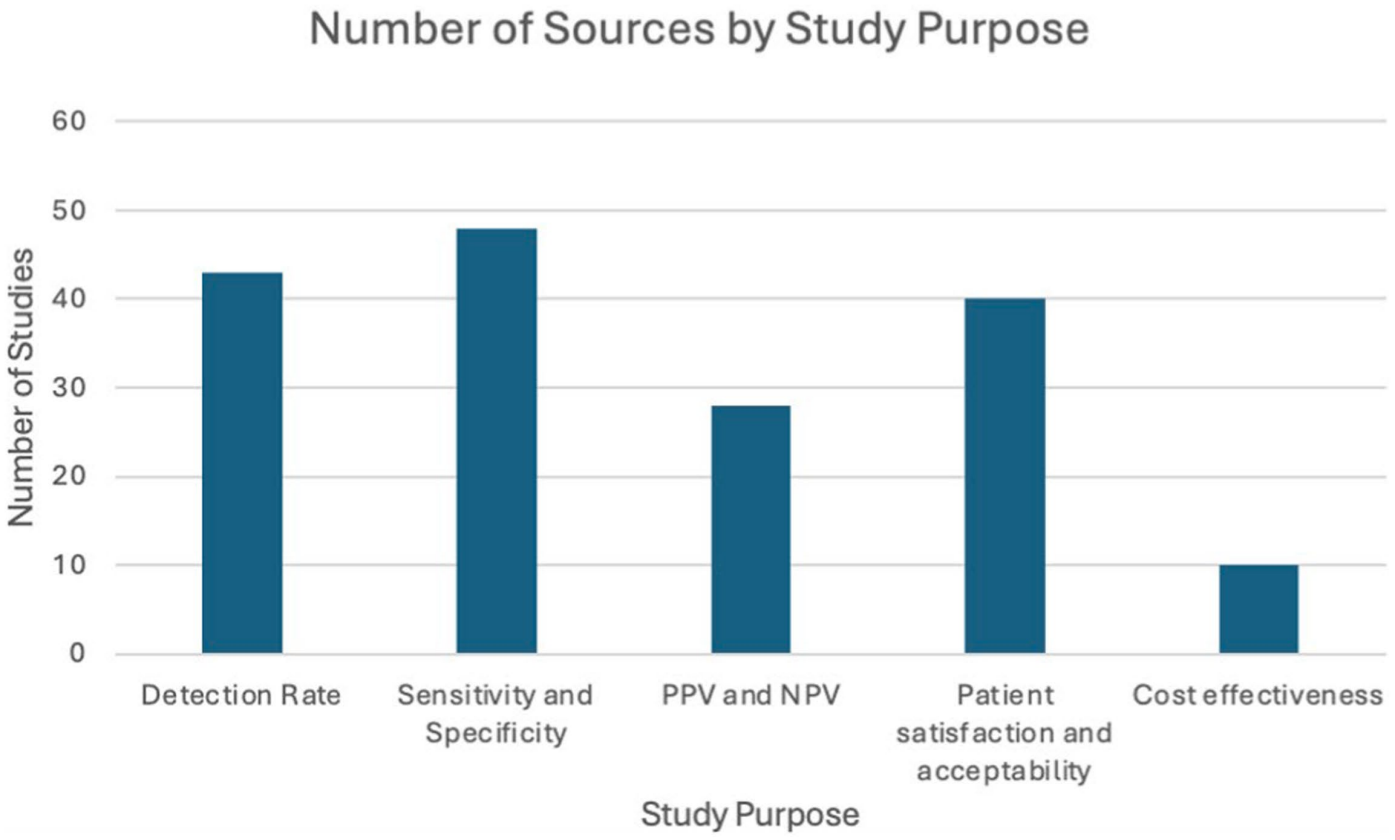
Distribution of included studies across key evaluation themes.

All included studies provided details on both self-sampling and clinician-collected sampling. The included studies were categorized into the following themes: detection rate, sensitivity and specificity, PPV and NPV, patient preference and acceptability, and cost-effectiveness as illustrated in the following bar chart (Figure 2).

### Statistical methods

#### Statistical test

A Wilcoxon signed-rank test was carried out to ascertain whether a significant difference was present between self-sampling and clinician-collected sampling in the examined themes. A critical value at alpha = 0.05 was obtained via a two-tailed test.

#### Area

Values incorporated in graphs were extracted exclusively from studies that were able to provide data for both self-sampling and clinician-collected sampling in the measured statistics (i.e., specificity, sensitivity, PPV, NPV, and patient preference). This was conducted to minimize bias in the comparison of self-sampling and clinician-collected sampling. An area graph was used to visualize the variability in the themes measured between self-sampling and clinician-collected sampling. An area estimate was calculated to help quantify the measures; (Y2 + Y1)/2*(X2-X1) for each point. The sum of all the points’ values was taken to give the overall area. The X change increments were kept constant at 1 for all points in all graphs. This is because we are not measuring area over another variable.

#### Comparative statistics

The agreement between self-sampling (SS) and clinician-collected sampling (CCS) methods for HPV detection was evaluated across various studies using kappa values. The average kappa value across these studies was 0.74, indicating strong agreement between the two methods. The highest kappa value reported was 0.88, indicating excellent agreement, while the lowest kappa value observed was 0.43, indicating moderate agreement but substantial variation in concordance between SS and PS methods (10, 11).

#### Statistical significance

When comparing self-sampling (SS) to clinician-collected sampling (CC) methods for HPV detection, the studies consistently found significant results. Seven studies reported *p*-values <0.001, showing the strong effectiveness of self-sampling. One study reported a strong agreement between self-sampling and clinician samples, with a *p*-value of <0.0001, highlighting the reliability of self-sampling (10).

While the majority of studies found strong support for self-sampling, some found no significant differences. For example, one study found a *p*-value of 0.527, showing no significant difference between the two methods.

### Detection rate

#### Detection rate in healthy patients

**Figure 3:**
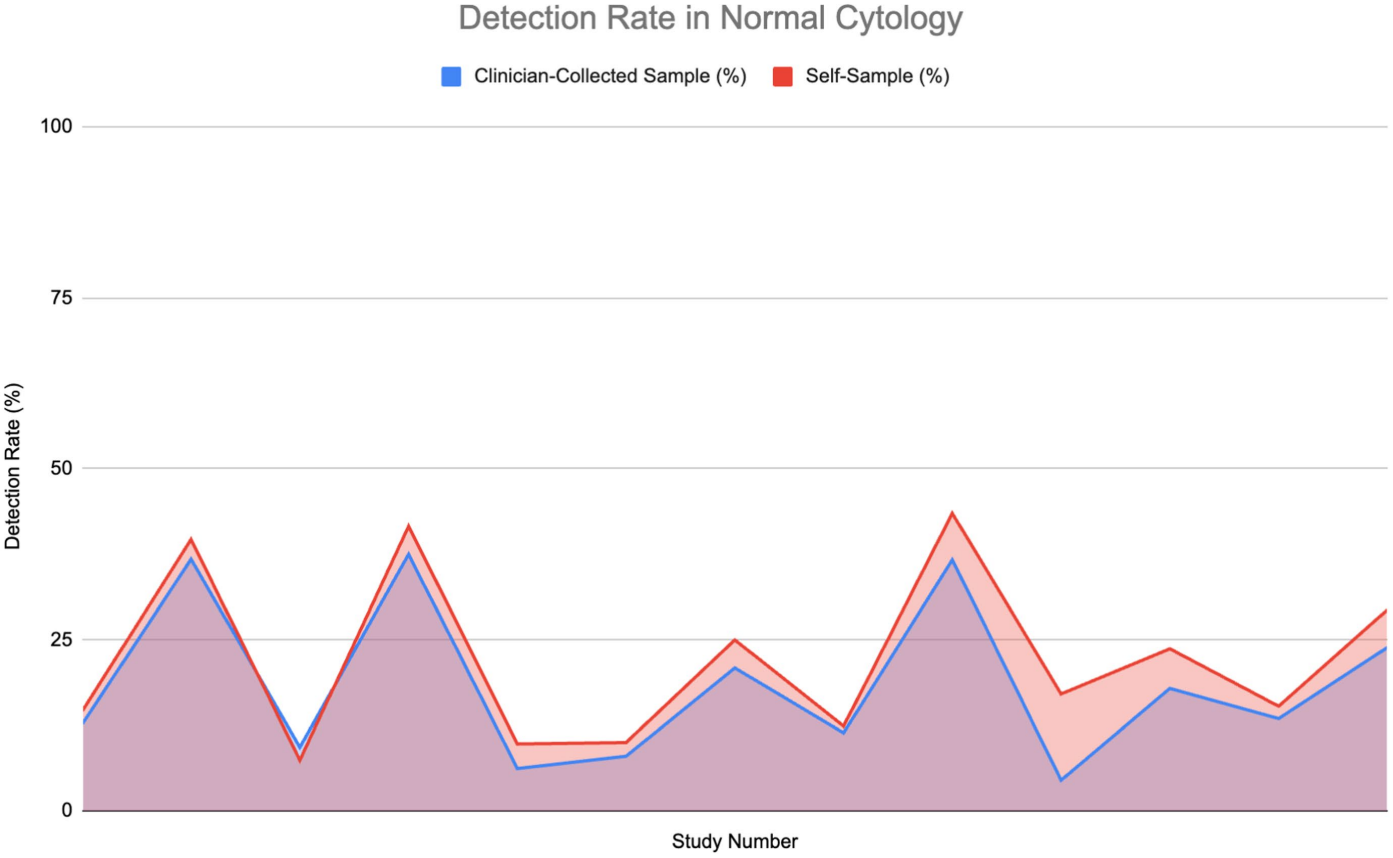
Comparison of HPV detection rates in healthy individuals: Self-sampling vs. clinician-collected sampling (20, 26, 32, 35, 36, 39, 43–50).

Figure 3 compares the HPV detection rates in normal, healthy individuals using self-sampling versus clinician-collected sampling across 14 studies. The graph indicates that self-sampling continuously detects a greater amount of HPV cases than clinician-collected sampling, with self-sampling detection rates reaching as high as 40% of tested individuals, whereas detection rates for clinician-collected sampling remain approximately 30%. In some cases, both methods show equivalent detection rates, especially when the rate is approximately 10%. At higher detection ranges, self-sampling consistently identifies a greater number of cases, indicating its potential to cover a wider range of HPV infections, especially those found in vaginal cells.

However, larger detection rates do not necessarily suggest increased test sensitivity. Sensitivity measures the ability of a test to accurately identify individuals truly infected with high-risk HPV types. In this study, a true positive case refers to an individual confirmed to be HPV-positive via validated molecular diagnostic techniques, such as PCR-based or hybrid capture tests, which are recognized for their accuracy in identifying high-risk HPV DNA. Although self-sampling identifies a greater number of total HPV cases, samples taken by clinicians show superior sensitivity to detecting HPV within populations with normal cytology. This is likely linked to the more focused method of clinician-collected cervical samples, which concentrate on the transformation zone of the cervix, where persistent high-risk HPV infections and precancerous lesions generally arise. The Wilcoxon signed-rank test established that although self-sampling identifies a greater number of HPV-positive cases overall, clinician-collected sampling has significantly superior sensitivity in detecting true positives in normal cytology cases.

Generally, the area under the self-sampling curve is bigger than that of the clinician-collected sampling, suggesting a more consistent and usually better performance throughout the studies for detecting HPV among normal, healthy individuals.

### Detection rate in patients with abnormal cytology

**Figure 4:**
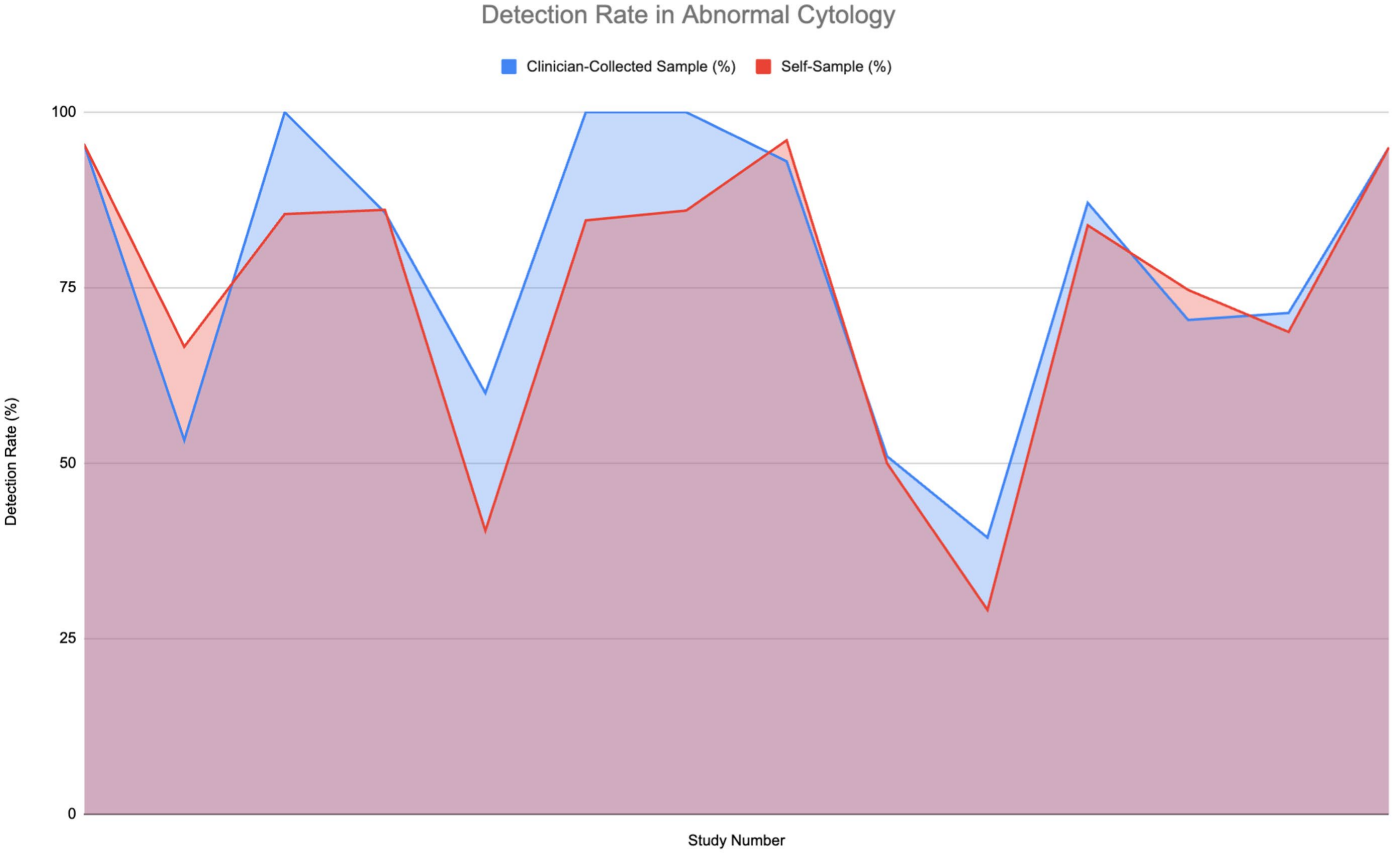
Comparison of HPV detection rates in individuals with abnormal cytology (6, 11–14, 27, 30, 33, 51–56).

Figure 4 illustrates the detection rates of HPV in patients with abnormal cytology or those who are HIV-positive, across 14 studies. This group includes various conditions such as cervical intraepithelial neoplasia 2+ (CIN2+), low-grade squamous intraepithelial lesion (LSIL), high-grade intraepithelial lesion (HSIL), atypical squamous cells of undetermined significance (ASCUS), and high-risk HPV (hrHPV).

Strong detection rates—reaching 75 to 100%—are shown by both self-sampling and clinician-collected sampling methods. Notably in the middle to high range, some studies find that self-sampling detection rates somewhat exceed those of clinician-collected sampling. For instance, in comparable settings, self-sampling can reach up to 85% or higher while detection rates for clinician-collected sampling might level off at 75%. A Wilcoxon signed-rank test was carried out to determine if a significant difference in sensitivity to normal cytology was present; however, the difference was found to not be significant.

The overall comparison of areas under the curves reveals that although clinician-collected sampling shows higher sensitivity, self-sampling attains high detection rates and serves as an efficient, accessible alternative for HPV screening.

### Sensitivity

#### Sensitivity in healthy patients

**Figure 5:**
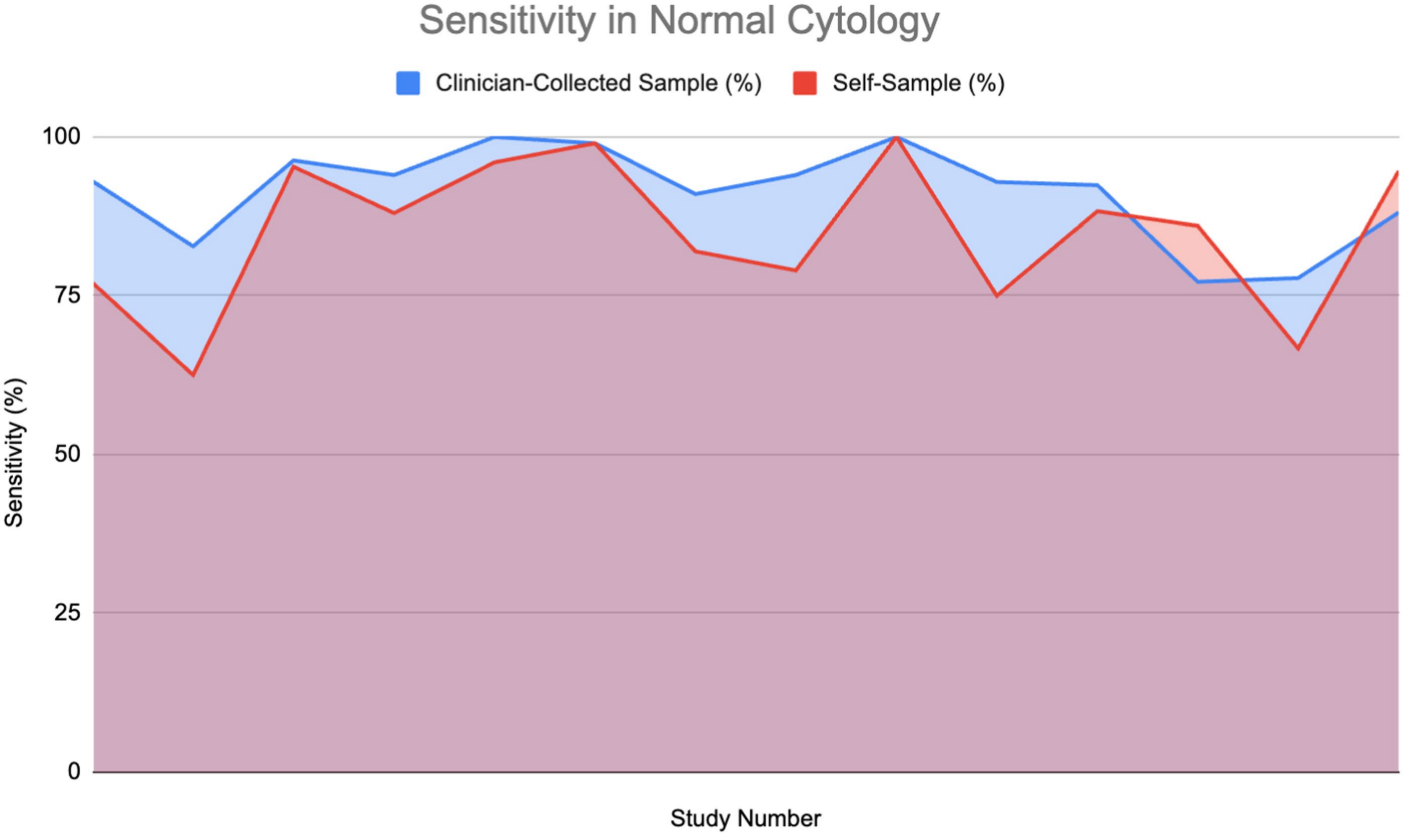
Sensitivity of HPV detection in healthy individuals using self-sampling vs. clinician sampling (13, 18, 24, 28, 29, 37, 40, 44, 48, 54, 57–59).

Figure 5 compares the sensitivity of HPV detection in normal, healthy patients using a self-sample for HPV testing against clinician-collected sampling over 14 studies. As shown in the area graph, both techniques have great sensitivity to HPV detection—between 75 and 100%. Notably for HPV testing, self-sampling often shows good performance, usually matching or even exceeding the HPV detection sensitivity of clinician-collected samples. The HPV detection sensitivity of self-sample and clinician-collected sampling was assessed using a Wilcoxon signed-rank test. The findings showed that although self-sampling for HPV testing is quite effective at identifying HPV infections, it is important to point out that it does not offer cytological examination. Therefore, clinician-collected samples remain essential for detecting cytological abnormalities such as HSIL or ASC-US.

Furthermore, studies including HIV-positive women, a group that is frequently at higher risk, revealed a sensitivity of 94.15% for self-sampling, indicating its strong reliability even in complex clinical conditions (10). The overlap of areas in the graph demonstrates that, while clinician-collected sampling occasionally has slightly higher sensitivity, self-sampling repeatedly performs at a comparable level, making it a reliable alternative for HPV screening in healthy people.

### Sensitivity in patients with abnormal cytology

**Figure 6:**
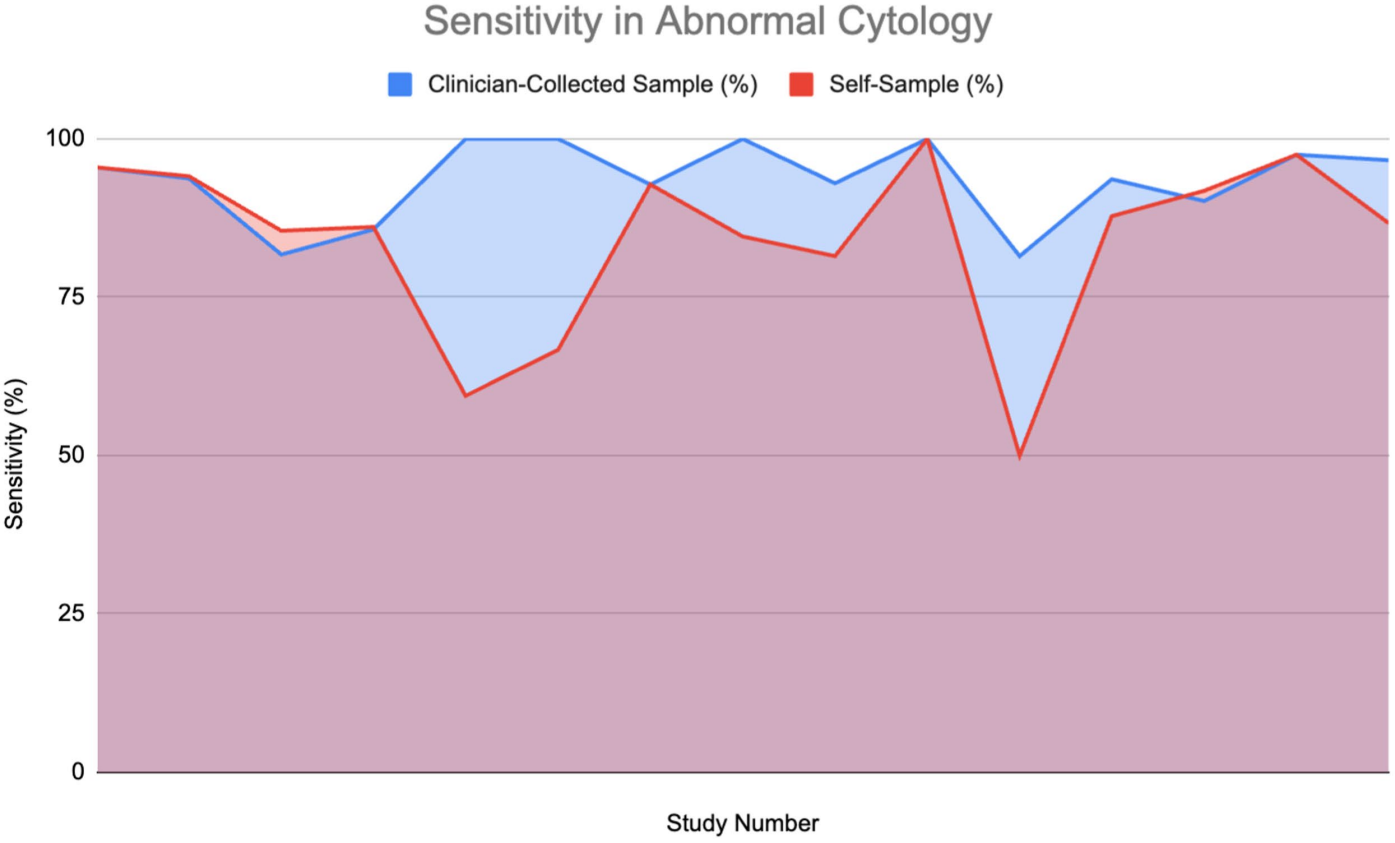
Sensitivity of HPV detection in patients with abnormal cytology (11, 12, 26, 27, 30, 33, 52–55, 60, 61).

Figure 6 shows the sensitivity of HPV detection in patients with abnormal cytology or who are HPV-positive across 15 studies. The findings show that self-sampling continues to demonstrate high sensitivity, frequently aligning with or closely matching that of clinician-collected sampling, with sensitivities generally ranging between 75 and 100%. A Wilcoxon signed-rank test (*W* = 10.0, *p* = 0.04086) confirmed that clinician-collected sampling has significantly higher sensitivity for detecting HPV in abnormal cytology cases. However, due to the minute difference in the graph, coupled with the lack of direct evidence detailing the sensitivity of self-sampling in abnormal cytology, we cannot conclude that self-sampling might not perform as efficaciously as clinician-collected sampling in HPV screening.

Four studies show that self-sampling for HPV testing and clinician-collected sampling had equivalent sensitivity rates, supporting the claim that self-sampling can be as effective as clinician-collected samples in detecting HPV, even in patients with complex conditions. Although clinician-collected sampling may show sensitivity benefits in particular situations, it is widely recognized that HPV testing provides greater sensitivity for identifying cervical precancer and cancer, especially when centered on high-risk HPV genotypes such as HPV 16 and HPV 18. The enhanced sensitivity of HPV testing is shown in the ability of self-sampling methods to achieve performance levels comparable to those of clinician-collected samples for HPV detection.

The graph indicates that, although clinician-collected samples can yield reliable results in certain situations, self-sampling for HPV testing is a reliable and efficient method for HPV detection, particularly in individuals with HIV-positive status, where increasing screening accessibility is critical. These findings further highlight HPV testing’s superior sensitivity for early cervical abnormality detection, reinforcing the role of self-sampling as a practical and scalable screening approach.

### Specificity

#### Specificity in healthy patients

**Figure 7:**
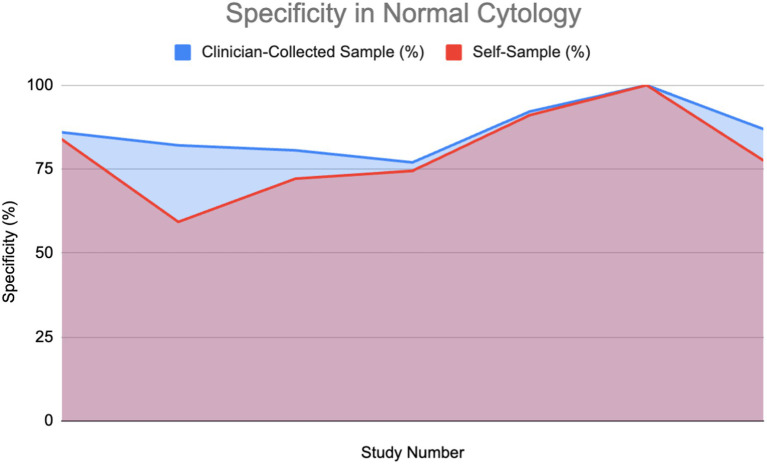
Specificity of HPV detection in healthy individuals: Self-sampling vs. clinician sampling (18, 24, 28, 37, 39, 40, 62).

Figure 7 shows specificity in healthy patients.

#### Specificity in patients with abnormal cytology

**Figure 8:**
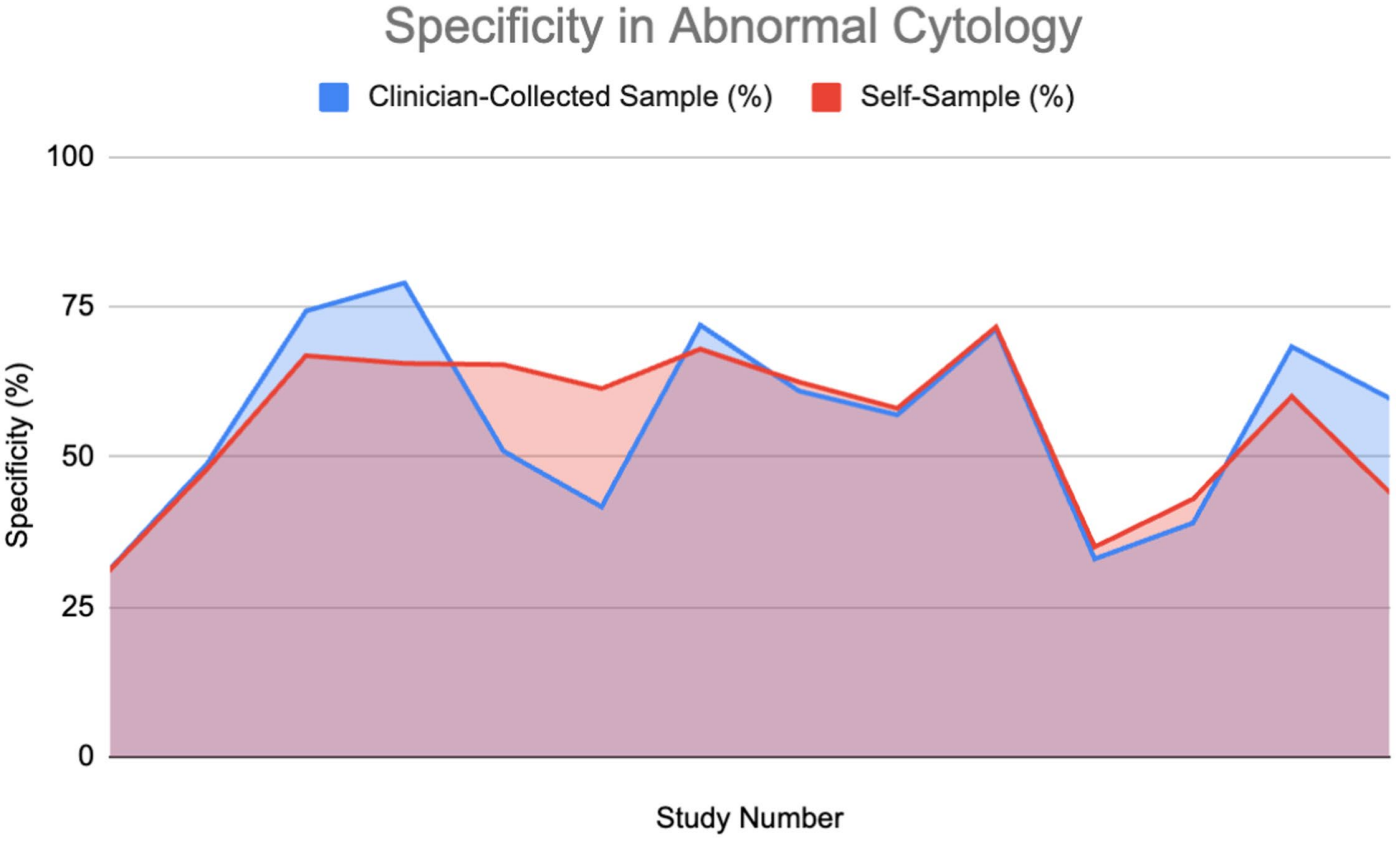
Specificity of HPV detection in patients with abnormal cytology (11, 14, 27, 30, 37, 52–54, 60–62).

A percentage of the papers were able to provide a direct value for specificity for both self-sampling and clinician-collected sampling (27%) detailing a direct comparison. These were used to plot area graphs for specificity in normal (Figure 7) and abnormal (Figure 8) cytology women. We have found a relationship between the values of specificity and whether the one testing is of abnormal or normal cytology; therefore, the graphs were split in two based on this. Of those papers that gave an indirect measure, most stated there is no significant difference in specificity between self-sampling and clinician-collected sampling (12–16).

In the abnormal cytology graph, the area was estimated to be 742.1 in SS and 743.3 in clinician-collected sampling, giving a ratio of approximately 1.002 with CS being 0.16% greater. For the normal cytology graph, the areas were estimated to be 518.35 and 477.86 in CS and SS respectively, giving an area ratio of 1.08, with CS being 8.4% greater. A Wilcoxon signed-rank test was carried out on both graphs. Normal cytology showed a significant difference in favor of clinician-collected sampling according to the Wilcoxon signed-rank test. The calculated test statistic W was calculated to be 0 and the *p*-value was found to be 0.02771 (<0.05), therefore we reject the null hypothesis. Abnormal cytology had mean percentages of 55.77 and 56.18% (SS and CS); evidence cannot conclude that a difference is significant.

### Positive predictive value (PPV) and negative predictive value (NPV)

#### PPV

Of the 30% that mentioned PPV, half gave a value for both clinician-collected and self-sampling methods, which were used to plot the area graph (Figure 8). There was no divide between abnormal and normal cytology as the results varied across both variables.

**Figure 9:**
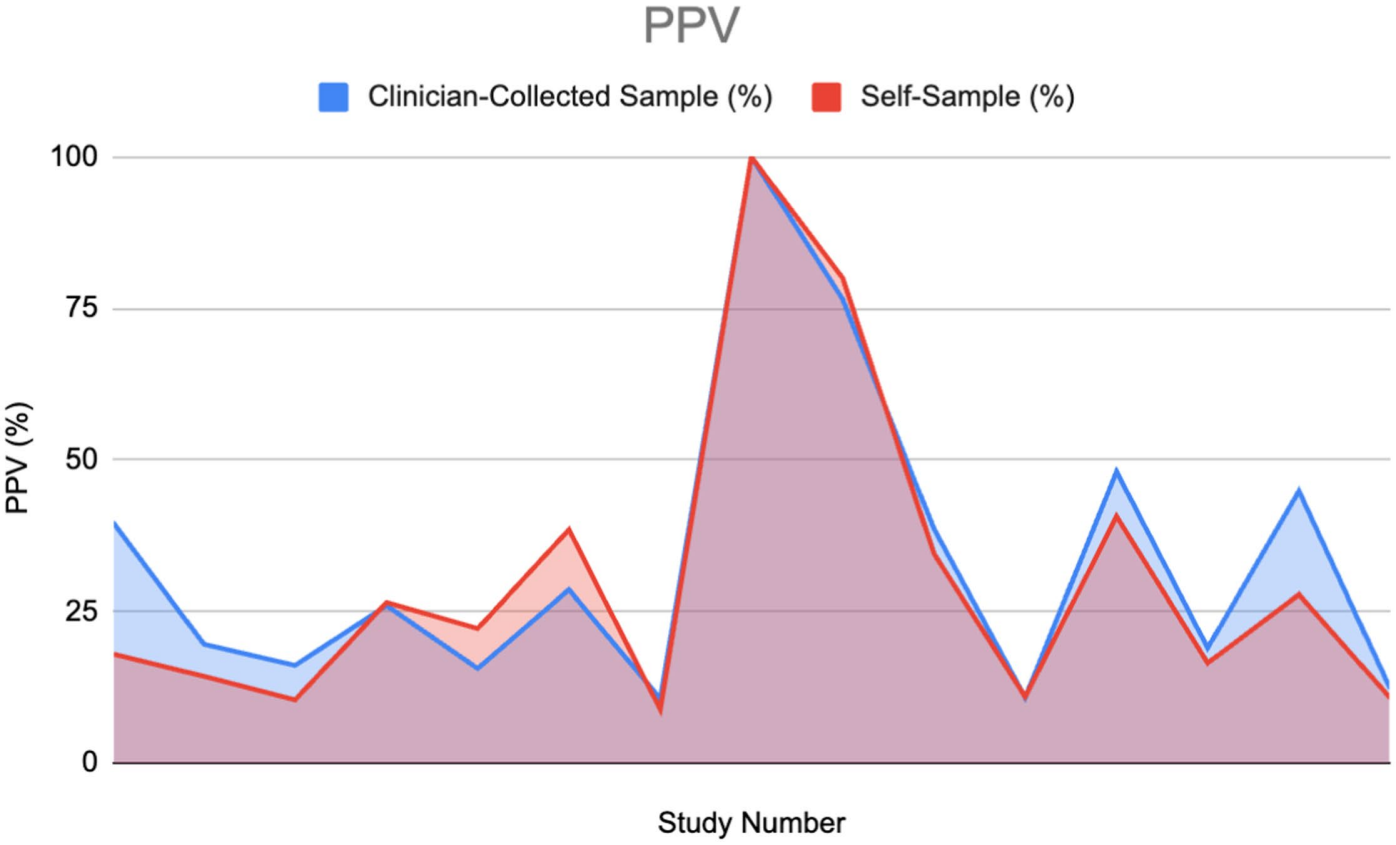
Positive predictive value (PPV) of HPV detection: Self-sampling vs. clinician sampling (11, 27–29, 33, 40, 46, 53, 54, 59–62).

PPV, similarly to NPV, would appear slightly higher overall in clinician-collected sampling than in self-sampling as shown in the graph. The estimated area calculated in CS and SS was 480.4 and 445.45 respectively, giving a ratio of 1.077 (CS 7.27% greater). Using the Wilcoxon signed-rank test, we cannot conclude a significant difference from the evidence provided (Figure 9).

#### NPV

The following area graph portrays a comparison between the negative predictive value (NPV) percentages of two different HPV diagnostic modalities: clinician-collected sampling and self-sampling.

High NPV in an HPV test is essential for clinical decision-making. When examining NPV in the included articles it was found that 15 of the studies gave values for NPV, while only 13 articles mentioned compared NPV of both self-sampling and clinician-collected sampling (2 of the 15 articles did not mention clinician-collected sampling NPV).

Using the Wilcoxon signed-rank test, no significant difference was found between the clinician-collected sampling NPV% and the Self Sampling NPV%. This insinuates that when solely considering NPV, self-sampling could be used as an alternative to clinician-collected sampling.

**Figure 10:**
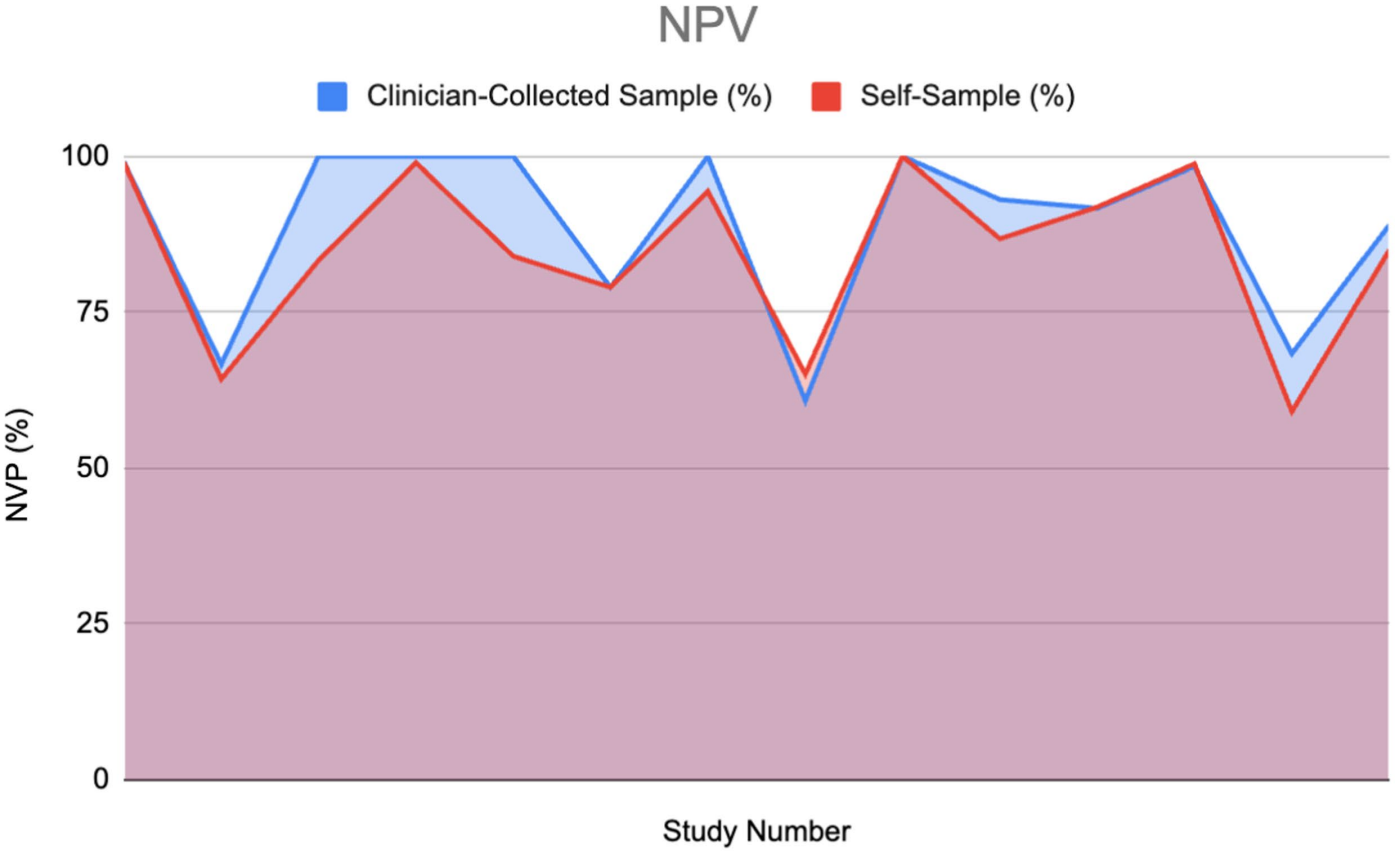
Negative predictive value (NPV) of HPV detection: Self-sampling vs. clinician sampling (13, 14, 22, 28, 30–38).

Figure 10 portrays a similar pattern with peaks and drops between both the self-sampling and clinician-collected sampling NPV, which portrays that their NPVs correlate. The total area under the clinician-collected sampling NPV curve is 1073.485, compared to 1025.69 for self-sampling. This gives a ratio of 1.047, indicating that overall, clinician-collected sampling has a slightly higher cumulative NPV compared to self-sampling. Furthermore, the ratio of 1.047 indicates that the difference between self-sampling and clinician-collected sampling is small and not clinically significant.

### Patient preference

**Figure 11:**
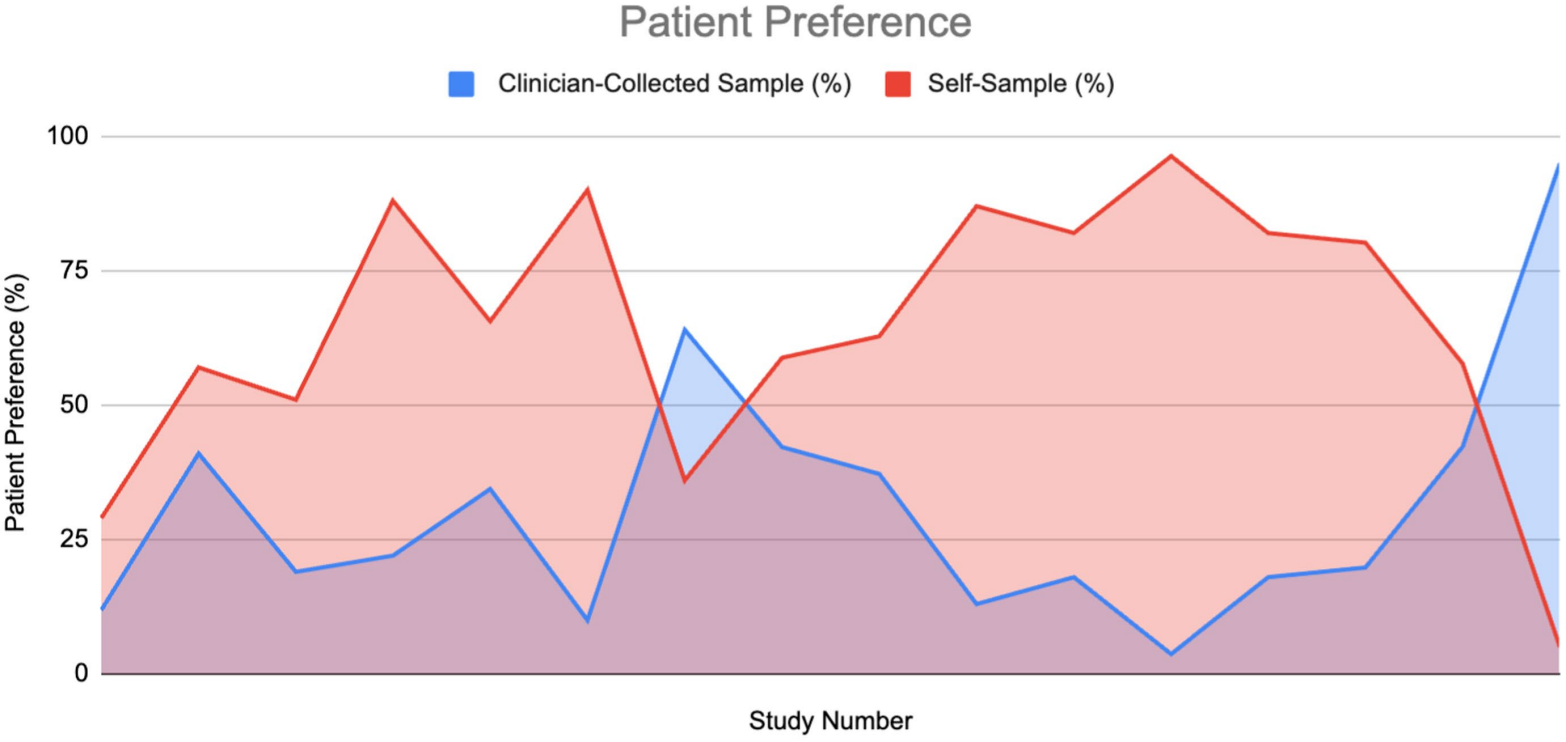
Patient preference for HPV sampling method: Self-sampling vs. clinician sampling (6, 10, 11, 20, 22, 35, 36, 38–40, 42, 62–66).

A total of 61% of the reviewed papers mentioned patient preference, with approximately 90% of these reporting positive data favoring self-sampling (SS) over clinician-collected sampling (CS). Among the studies that provided specific percentages for women’s preferences between SS and CS, we plotted an area graph to visualize the overall preference distribution (Figure 11). For papers that reported a preference for SS but did not specify a corresponding value for CS, we assumed the remainder of the preference (i.e., 100% minus the reported SS preference) was attributed to CS.

As illustrated in the graph, the area under the curve for self-sampling is substantially larger, indicating a greater overall preference for SS compared to CS (1011.4 vs. 438.05). The ratio of these areas was approximately 2.3. The mean preference values were 64.3% for SS and 30.7% for CS. A Wilcoxon signed-rank test confirmed that this difference was statistically significant, providing strong evidence in favor of self-sampling. Additionally, 84% of studies that did not report specific preference values still indicated a preference for SS, citing factors such as ease of use, comfort, reduced embarrassment, less pain, and higher acceptability.

### Cost-effectiveness

Ten articles explicitly discussed the cost-effectiveness of self-sampling versus clinician-collected sampling. Of these, six presented general conclusions on the topic, and four reported specific cost-effectiveness values. These studies spanned 10 different countries, all of which concluded that self-sampling is more cost-effective than clinician-collected sampling.

#### Overall findings

Self-sampling was consistently found to be more cost-effective than clinician-collected sampling in all ten investigations, indicating that self-sampling may be a better choice when costs are considered.

#### Prominent research

*[China]*: In rural and urban environments, self-sampling produced the largest increases in quality-adjusted life years (QALYs), ranging from 220 to 440 QALYs. Self-sampling strategies were shown to be more cost-effective than current methods, with savings between $3,540 and $818,430. On the other hand, the total expense of the clinician-collected sampling was higher, with additional costs ranging from $20,840 to $182,840, suggesting that self-sampling would be the best option (17)*. [United States]:* According to this study, self-sampling was more effective in reducing the lifetime risk of cervical cancer by 14.8%, compared to a 6.4% reduction for clinician-collected sampling. Additionally, self-sampling costs $62,720 for every year of life saved (YLS) (18)*. [Sweden]:* According to this study, clinician-collected sampling costs at least €40, whereas self-sampling devices cost less than one-tenth of that amount (14). *[France]:* This research showed that self-sampling was cost-effective, with an incremental cost-effectiveness ratio (ICER) of €63.20 per additional screened woman compared to no intervention (19).

## Discussion

This systematic review aimed to evaluate the effectiveness of self-sample screening methods for HPV compared to traditional clinician-collected sampling, the longstanding gold standard in cervical cancer screening. There has been a substantial amount of research dedicated to comparing self-sampling with clinician-collected samples for HPV detection. The onset of the COVID-19 pandemic further highlighted the need for alternative screening methods as healthcare systems worldwide faced unprecedented challenges. Self-sampling gained increased attention during this period as a potential solution to maintaining screening efforts while minimizing in-person visits. The global health crisis underscored the importance of self-administered screening methods, with self-sampling emerging as a feasible option for maintaining continuity in HPV screening programs amidst lockdowns and healthcare disruptions.

Our review synthesizes data from numerous studies, collectively suggesting that self-sampling for HPV detection is as effective as clinician-collected sampling, with minimal differences observed in HPV detection rates. This consistency across studies reinforces the potential for self-sampling as a reliable screening tool. These results have significant implications for public health, particularly in expanding screening access to underserved populations. This discussion will explore the considerations and limitations of the existing evidence, as well as provide direction for future research.

### Summary of key findings

#### Detection rate and sensitivity

Our review consistently found that self-sampling is comparable to clinician-collected sampling in terms of HPV detection rates and sensitivity, supporting the idea that self-sampling can be a viable alternative to cervical cancer screening. Across the studies, self-sampling showed comparable or greater effectiveness than clinician-collected sampling in detecting HPV in individuals without any pre-existing health issues or in populations with greater susceptibility. For instance, in one of the studies focused on HPV detection in human immunodeficiency virus (HIV)-positive women, self-sampling detected HPV in 43.50% of cases, compared to 36.70% with clinician-collected sampling (20). Another study found similar results, with self-sampling detecting 29.40% of HPV cases, while clinician-collected sampling detecting 23.90% (21). These findings suggest that self-sampling may have a higher detection rate for HPV in certain high-risk populations, such as women with HIV. However, this does not necessarily suggest that it has more sensitivity to identifying clinically relevant HPV infections. This highlights the capacity of self-sampling to be similarly effective at identifying HPV, particularly in groups with a higher risk.

In individuals with abnormal cytology or who are HPV-positive, both methods had high detection rates, ranging from 50 to 100%. Overall, the results show that self-sampling works well, often as well as clinician-collected sampling, especially when it comes to finding high-risk HPV types. A study revealed that, while self-sampling was generally effective, its detection rate for CIN3+ cases was inferior to that of clinician sampling (22). This mismatch may be linked to differences in anatomical sampling and HPV genotype distribution. Self-sampling primarily gathers vaginal cells, while clinician-collected specimens specifically focus on the cervix, particularly the transformation zone, where high-grade lesions are most susceptible to arise (23). Therefore, self-sampling may inadequately identify CIN3+ lesions, resulting in lower sensitivity to detecting certain abnormalities (24). Certain high-risk HPV genotypes, including HPV 16 and HPV 18, show a greater affinity for cervical epithelial cells, increasing their likelihood of detection in clinician-collected samples (25).

In a study focused on identifying HSIL in healthy persons, self-sampling for HPV testing obtained a perfect sensitivity of 100%, while clinician-collected cytology detected only 45% of ASC-US+ cases (26). This significant finding shows that self-sampling for HPV testing can detect high-risk HPV infections associated with precancerous lesions with the same efficacy as conventional clinician-collected cytological analysis.

The constancy of sensitivity across studies is backed by statistically significant results. Seven studies found *p*-values <0.001, indicating the reliability of self-sampling as a diagnostic method for HPV. One study found significant agreement between self-sampling and clinician-collected sampling, with a kappa value of 0.88 and a *p*-value of <0.0001, indicating strong concordance between the two methods. Moreover, in trials involving HIV-positive patients within the abnormal cytology group, self-sampling for HPV testing showed a sensitivity of 92.6%, emphasizing its efficacy across various patient populations (10). These findings are important for ongoing efforts to enhance cervical cancer screening in regions with limited resources. Self-sampling provides a convenient and patient-friendly method of screening that maintains diagnostic accuracy.

#### Specificity

As mentioned in the specificity results section in more detail, we found a little increase in specificity between CS and SS across both graphs. Normal cytology showed a slight significant difference in favor of clinician-collected sampling. Abnormal cytology differences were found to be insignificant.

Aiko KY et al. (abnormal cytology) provide the strongest evidence supporting the specificity of self-sampling, demonstrating a 20% difference in specificity between self-sampling (SS) and clinician sampling (CS) (27). In the comparative study, 159 participants were enrolled, with 136 completing the study, suggesting relatively weak reliability. The CASP checklist confirmed good validity.

In contrast, Mremi A et al. presented the weakest evidence for SS compared to CS (82.1% vs. 59.3%) (28). This combined cross-sectional study included 1,620 women, indicating stronger reliability. The paper also demonstrated good validity according to the CASP checklist.

The presence of studies illustrating significantly higher specificity in SS suggests that the difference in specificity may not be significant. As a result, for women who prioritize comfort, less embarrassment, and less pain, the significant difference in preference could outweigh the small difference in specificity. This implies that more women may choose self-sampling generally, as they are less likely to be deterred by the negative effects of the slight decrease in specificity.

#### PPV

As mentioned in the PPV results section, we have found there to be little difference between the PPV of self-sampling and clinician-collected sampling; we determined this difference is statistically insignificant.

Fujita M et al. provide the strongest evidence for PPV in self-sampling compared to clinician-collected sampling (38.5–28.6%) (29). The paper used a high sampling number: 7337 for self-sampling and 7,772 for clinician-collected sampling. This indicates good reliability. The paper was also evaluated with CASP and risk bias (ROB) which was shown to have good validity and low bias. In contrast, Mremi A et al., in a cross-sectional study in Tanzania, present the strongest increase in PPV for clinician-collected sampling compared to SS (39.8–18%) (28). The CASP checklist showed good validity. A weakness of this paper is the low sampling amount, 1,620, which makes the results less reliable than the previous paper mentioned.

Certain articles displayed in the Results section showed instances of PPV in self-sampling being higher than clinician-collected sampling. This suggests that, for women who prioritize comfort, reduced embarrassment, or less pain, the strong preference for self-sampling may outweigh the small differences in PPV and specificity. As a result, self-sampling could lead to greater overall participation in HPV screening.

#### NPV

The processing and analysis of the data within the included studies revealed variability in the comparison between the negative predictive values (NPVs) of self-sampling and clinician-collected sampling. In three studies, self-sampling demonstrated a higher NPV than clinician-collected sampling, with differences ranging from 0.2 to 4.3% (13, 30, 31). Two studies reported equal NPVs for both methods (32, 33). In three other studies, self-sampling had a slightly lower NPV than clinician-collected sampling, with differences ranging from 0.09 to 2.4% (22, 28, 34). In the remaining five studies, self-sampling had significantly lower NPVs than clinician-collected sampling, with differences ranging from 5.9 to 16.7% (14, 35–38).

The disparity in the results observed in Figure 10 could be explained by the different types of self-sampling kits or cytology used. For instance, the largest difference between the self-sampling NPV values was 39.98%, wherein one used the Evalyn Brush and the other used a standard flock tip swab (22, 37). Additional research may be required to explore further confounding variables that led to this disparity.

The mean NPV percentages for self-sampling and clinician-collected sampling were 85.46 and 84.98%, respectively, suggesting that, on average, the NPVs of both methods are comparable. However, the total area under the NPV curve was slightly higher for clinician-collected sampling (1073.485) compared to self-sampling (1025.69), resulting in a ratio of 1.047. This ratio indicates that overall, clinician-collected sampling has a marginally higher cumulative NPV than self-sampling. These differences are minimal and do not appear clinically significant, suggesting that the difference in NPV between them is unlikely to impact clinical outcomes or decision-making processes. Therefore, this suggests that self-sampling can be used as an alternative to cytology.

#### Patient preference

As detailed in the Results section, self-sampling was strongly preferred over clinician-collected sampling, providing robust evidence of a significant difference.

The strongest evidence supporting SS over CS in terms of preference was reported by Des Marais et al., with 96.3% of participants favoring SS compared to 3.7% for CS (39). However, as this observational study included only 193 women, its reliability is somewhat limited. Cochrane ROB and CASP assessments indicated a low risk of bias and good validity. Conversely, the weakest evidence for SS preference came from a cross-sectional study by Ramesan et al., where only 5% of participants preferred SS over CS (95%) (40). This study, which enrolled just 42 women, had even lower reliability than that of Des Marais et al. Despite the low preference for SS in this study, all participants (100%) found SS acceptable, with 95% reporting that it was neither embarrassing nor painful. Cochrane ROB and CASP evaluations also indicated a low risk of bias and good validity for this study.

The significant difference in this domain is drastically greater than the significant differences measured within the other factors measured (PPV, specificity, etc.). The preference factor can be a major cause of women testing than not testing at all; and with the recorded benefits of self-sampling, it would seem this to be a better option that leads to overall more women screening for cervical cancer. Women who prefer self-sampling due to comfort, less pain, less embarrassment, etc., may ultimately negate the small differences in specificity observed with clinician-collected sampling (e.g., a 6.6% difference in means). The more tests conducted, the less likely a false negative will occur. Additionally, while many women feel more comfortable using self-testing options, it is important to provide support to ensure that they can self-test confidently and appropriately (41).

#### Cost-effectiveness

Different factors contribute to determining the cost-effectiveness of different screening modalities; the main ones entail cost of intervention, effectiveness of intervention, and country/healthcare system.

Cost of intervention: Of the 10 studies, 3 explicitly mentioned the cost of self-sampling and clinician-collected sampling, which indicated that self-sampling is the optimal choice (14, 17, 19).

Effectiveness of Intervention: This mainly considers clinical effectiveness and health outcome metrics such as quality-adjusted life years (QALYs). A study in the United States delved into this and explained that self-sampling was more effective in reducing the lifetime risk of cervical cancer by 14.8%, compared to a 6.4% reduction for clinician-collected sampling (18).

Country/Healthcare system: Different countries/hospitals may have different healthcare systems or screening strategies, which would explain the difference in the values for the cost of intervention, as each study was based in a different country [China, Sweden, and France] (14, 17, 19).

### Comparison with existing literature

The findings of this systematic review align with a growing body of literature investigating the effectiveness of self-sampling HPV screening. Multiple studies have consistently reported comparable results between self-sampling and clinician-collected sampling in terms of HPV detection rates, sensitivity, and specificity. For instance, one study concluded that self-sampling is non-inferior to clinician-collected sampling, particularly in high-risk populations, a result echoed in the findings of our review (24). Similarly, another study demonstrated that self-sampling and clinician-sampling for HPV have equivalent sensitivity for detecting high-grade cervical intraepithelial neoplasia (CIN2+), which is consistent with the results of our systematic review (13). These studies reinforce the conclusion that self-sampling can be as effective as traditional methods, especially for increasing coverage in under-screened populations.

Additionally, several studies have highlighted the practicality and convenience of self-sampling, which may improve patient adherence to screening programs. For example, one study reported that self-sampling was particularly well received in rural and underserved communities, which aligns with the findings from our review that suggest self-sampling can help bridge the gap between existing healthcare services and their accessibility to people of different demographic backgrounds (42). However, our review also uncovered some variability in the concordance between self-sampling and clinician-collected sampling, particularly in detecting more severe lesions (e.g., CIN3+). This observation is consistent with a study that suggested that while self-sampling performs comparably well, there still may be a slight edge to clinician-collected samples for detecting the most severe abnormalities (43).

Finally, it is important to mention the increased attention to self-sampling during the COVID-19 pandemic, which further propelled its adoption, as highlighted in other recent systematic reviews. Studies published during the pandemic emphasized the crucial role of self-sampling in maintaining screening rates while reducing the need for in-person clinic visits (18). This mirrors the findings from our review, where self-sampling was shown to be a viable option for expanding screening accessibility during times of strain on the healthcare system.

### Implications for practice, policy, and further research

The results of this review have significant implications for both clinical practice and healthcare policy. First, evidence supporting the comparable efficacy of self-sampling and clinician-collected sampling for HPV detection suggests that self-sampling could be incorporated more widely into cervical cancer screening programs. This could help address barriers to screening, such as limited access to healthcare facilities, patient discomfort with clinician-administered tests, and the need for frequent clinical visits. Policymakers should consider promoting self-sampling as an option, particularly for under-screened or hard-to-reach populations, as it has the potential to increase screening uptake and reduce cervical cancer incidence.

From a policy perspective, increasing access to self-sampling kits through public health initiatives or partnerships with community organizations could further enhance participation rates. Programs in countries including Sweden and the Netherlands have already demonstrated the feasibility and cost-effectiveness of offering self-sampling kits as part of national screening programs. Scaling up similar models in other countries could lead to a more inclusive approach to cervical cancer prevention, reducing the disparity in screening coverage across different socioeconomic and geographic populations.

For clinical practice, the potential integration of self-sampling into routine HPV screening protocols would necessitate clear guidelines and education for both patients and healthcare providers. Patient education materials should address concerns about sample accuracy and explain how to properly perform self-sampling to ensure reliable results. Moreover, clinicians should be trained to support patients who choose self-sampling, offering guidance on follow-up procedures in the event of a positive result.

Regarding future research, long-term studies are needed to assess the performance of self-sampling over multiple screening rounds to determine its true effectiveness in reducing cervical cancer incidence. Additionally, more research is required to evaluate the sensitivity of self-sampling in detecting high-grade lesions such as CIN3+, given that the limited short-term evidence has not been promising thus far. In that same regard, longer-term studies looking into cost-effectiveness, patient preference, and patient adherence would also be beneficial in giving us a more well-rounded understanding of the true efficacy of self-sampling methods in cervical cancer detection.

### Limitations

Several factors impact the validity of comparisons made using the graphs, stemming from variables such as: (1) the type of brush/self-sampling method used (where multiple were mentioned, an average was calculated), (2) the type of diagnostic assays used, (3) the type of neoplasia assessed (CIN2, CIN3, HSIL, and ASC-US), (4) the health status of the women tested (abnormal/normal cytology and HIV positive/negative), and (5) the timeline of when the test was conducted.

The lack of sufficient datasets to provide a more precise area estimate and percentage increase: 13 datasets were averaged for CS and SS for each graph.

We found it was not possible to use a parametric method to determine the significance between clinician-collected sampling and self-sampling within each theme. This was because the samples were low for each measure, making it harder to test for normality.

## Conclusion

This systematic study found that self-sampling HPV screening is safe and reliable, with equivalent detection rates and sensitivity to clinician-collected sampling. Across a range of trials, self-sampling revealed comparable, and in some cases, superior outcomes, particularly in high-risk populations such as HIV-positive women. The concordance in diagnostic performance between self-sampling and clinician-collected sampling highlights the possibility of self-sampling being broadly embraced as a key screening method for cervical cancer, therefore helping to address gaps in healthcare access and improve early detection outcomes.

Self-sampling was also the preferred method among women, with studies indicating a strong preference for its convenience, privacy, and ease of use. This comfort and acceptability as well as its proven cost-effectiveness point to self-sampling’s potential to raise national screening program participation rates, especially among underprivileged groups. The economic benefits of lower screening costs further support the case for integrating self-sampling into public health strategies aimed at reducing the burden of cervical cancer.

Although the results are supportive, several variations in self-sampling techniques call for more research. Continued research is needed to ensure consistency in screening results across diverse populations and to optimize the effectiveness of self-sampling in long-term cervical cancer prevention efforts. Currently, extensive research is being conducted on HPV genotyping to enhance the detection of HSIL and its progression to cervical cancer. Still, the review emphasizes the important part self-sampling can play in increasing public health outcomes worldwide and therefore widening access to cervical cancer screening.

## Data Availability

The original contributions presented in the study are included in the article/Supplementary material, further inquiries can be directed to the corresponding author.
